# The Location of Missense Variants in the Human GIP Gene Is Indicative for Natural Selection

**DOI:** 10.3389/fendo.2022.891586

**Published:** 2022-06-29

**Authors:** Peter Lindquist, Lærke Smidt Gasbjerg, Jacek Mokrosinski, Jens Juul Holst, Alexander Sebastian Hauser, Mette Marie Rosenkilde

**Affiliations:** ^1^Laboratory for Molecular Pharmacology, Department of Biomedical Sciences, Faculty of Health and Medical Sciences, University of Copenhagen, Copenhagen, Denmark; ^2^Novo Nordisk Research Center Indianapolis, Indianapolis, IN, United States; ^3^Department of Biomedical Sciences, Faculty of Health and Medical Sciences, University of Copenhagen, Copenhagen, Denmark; ^4^Novo Nordisk Foundation Center for Basic Metabolic Research, Faculty of Health and Medical Sciences, University of Copenhagen, Copenhagen, Denmark; ^5^Department of Drug Design and Pharmacology, Faculty of Health and Medical Sciences, University of Copenhagen, Copenhagen, Denmark

**Keywords:** GIP - glucose-dependent insulinotropic peptide, missense variants, pharmacogenomics, GIPR, GPCR (G protein coupled receptor), UK Biobank

## Abstract

The intestinal hormone, glucose-dependent insulinotropic polypeptide (GIP), is involved in important physiological functions, including postprandial blood glucose homeostasis, bone remodeling, and lipid metabolism. While mutations leading to physiological changes can be identified in large-scale sequencing, no systematic investigation of GIP missense variants has been performed. Here, we identified 168 naturally occurring missense variants in the human GIP genes from three independent cohorts comprising ~720,000 individuals. We examined amino acid changing variants scattered across the pre-pro-GIP peptide using *in silico* effect predictions, which revealed that the sequence of the fully processed GIP hormone is more protected against mutations than the rest of the precursor protein. Thus, we observed a highly species-orthologous and population-specific conservation of the GIP peptide sequence, suggestive of evolutionary constraints to preserve the GIP peptide sequence. Elucidating the mutational landscape of GIP variants and how they affect the structural and functional architecture of GIP can aid future biological characterization and clinical translation.

## Introduction

Glucose-dependent insulinotropic polypeptide (originally: gastric inhibitory polypeptide) (GIP) is a peptide hormone of 42 amino acids secreted from intestinal K cells in response to intake of nutrients (1). Like glucagon-like peptide-1 (GLP-1), GIP is an incretin hormone that postprandially potentiates glucose-induced insulin secretion from pancreatic β-cells (2–4). In patients with type 2 diabetes (T2D), the incretin effect is impaired (4), partly due to a reduction in GIP efficacy (5). The proposed roles of GIP in various physiological functions, including lipid metabolism and bone remodeling, has intensified the investigations of the GIP system and its therapeutic potential (6–10). The GIPR is expressed in human adipose tissues (11), and high levels of circulating GIP are associated with high body mass index (BMI) (12), further supported by a GIPR knock-out mouse model, which is resistant to high fat diet-induced obesity (1, 13). An increase in bone formation and decrease in bone resorption markers upon GIP administration suggest a role for GIP in bone remodeling (14–16). Supporting this, administration of the selective GIPR antagonist GIP(3-30)NH_2_ resulted in inhibition of GIP actions on the bone cells (6, 7, 14–16). In contrast to the reduced insulinotropic actions of GIP in patients with T2D, the suppression of bone resorption by endogenous GIP seems conserved in patients with T2D (17). Supporting an important role for GIP in bone remodeling, mutations in the GIPR have been associated with increased fracture risk and decreased bone mineral density (18).

**Figure 1:**
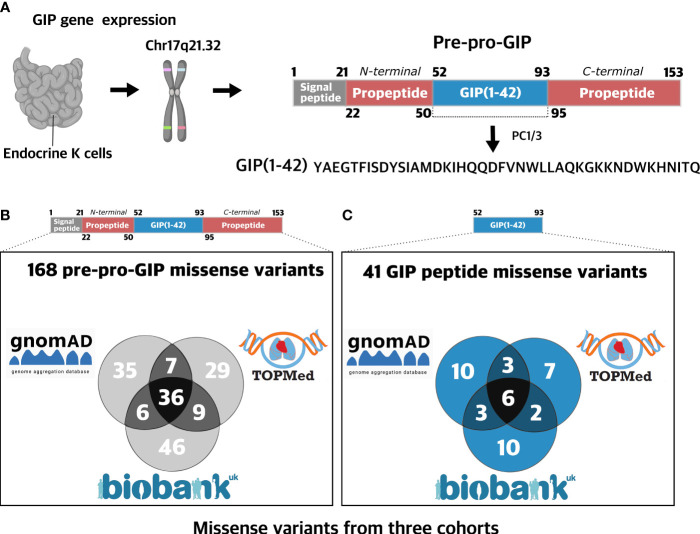
Prevalence of missense variants in the GIP gene. **(A)** The human glucose-dependent insulinotropic polypeptide (GIP) gene is located on chromosome 17q21.32 and is mainly expressed in the stomach and in K cells of the small intestine epithelium (1). The GIP peptide is derived from a 153 amino acid prohormone, pre-pro-GIP, encoding four domains: a signal peptide (1-21), an N-terminal propeptide fragment (22-50), GIP(52-93), and a C-terminal propeptide fragment (95-153). Intracellularly, GIP(1-42) is liberated from the prohormone upon processing *via* PC1/3 cleaving at single arginine residues (19). **(B)** 168 missense GIP gene variants were aggregated from three independent cohorts with a total of 721,991 participants: UK Biobank (454,787 exomes) (20), gnomAD (125,748 exomes and 15,708 genomes) (21), and TOPMed (132,345 exomes) (22). The variants are found in 105 different amino acid positions (69%). **(C)** 41 missense variants identified in the GIP gene were found in the sequence encoding the biologically active GIP(1-42) peptide spanning 24 different amino acid positions (57%). **Figure 1A** Created with BioRender.com.

The GIP gene is located on chromosome 17q21.32 and encodes the 153 amino acid prohormone, pre-pro-GIP, which is composed of the biologically active GIP peptide (also denoted GIP(1-42); a 21 amino-acid long signal peptide; and an N-terminal and a C-terminal propeptide fragment (**Figure 1A**) (23). In the post-translational process, prohormone convertase (PC) 1/3 which cleaves after dibasic amino acid motifs, or at single arginine residues, liberates the biologically active GIP(1-42) peptide from the precursor (19). GIP(1-42) is a target for the ubiquitous enzyme DPP-4 (dipeptidyl peptidase 4), which cleaves GIP at the alanine residue in position 2, resulting in the formation of the inactive metabolite GIP(3-42) (24). The metabolite GIP(3-42) acts as a weak GIPR antagonist, and inhibits the insulinotropic effect of GIP(1-42) when present at high (supraphysiological) levels (25). A C-terminally truncated GIP peptide, GIP(1-30)NH_2_, has also been identified in the circulation at low concentrations. It is presumably derived from the gut and acts as a full GIPR agonist *in vitro* and *in vivo* (6, 26–28). In addition to these well-characterized N- and C-terminally truncated variants, another fragment of the pre-pro-GIP precursor, GIP_HUMAN[22-51], was recently discovered. It overlaps with the N-terminal propeptide fragment and was suggested to possess pro-atherosclerotic effects (29).

GIP(1-42) appears to signal through a single receptor, the GIPR, which is a Gα_s_ coupled receptor, activating adenylyl cyclase, resulting in generation of cAMP with subsequent downstream signaling (30, 31). Moreover, the GIPR has been shown to also signal through Gα_i_ and Gα_q_ to some extent (32, 33). The GIPR belongs to the class B1 (secretin-like) G protein-coupled receptors (GPCR) well known for their large N-termini, important for initial ligand binding. According to the “two-domain” model, the receptor N-terminus recognizes and binds the C-terminal region of the peptide hormone which, subsequently, allows docking of the N-terminal region of the ligand into the receptor binding pocket formed by transmembrane (TM) domains; this leads to receptor activation (34, 35). In addition to the endocrine pancreas, the GIPR is expressed in additional tissues including the heart, bones, several brain regions, adipose tissue as well as the gut (36). Recently, two naturally occurring missense variants in the GIPR gene were described to be associated with a lower body mass index (BMI) in human carriers. *In vitro* studies indicate that these GIPR variants result in reduced G protein coupling and impaired β-arrestin 2 recruitment, conceivably providing a molecular explanation for the reduced body weight phenotype (37). However, to date, no systematic investigations of human variants in the proGIP gene or the region encoding GIP (1-42) have been conducted. Pharmacological characterization of several truncated GIP peptides has supported the pivotal role of the N-terminus for receptor activation, consistent with the generally accepted activation mechanism of class B1 receptors (6, 35). Based on the knowledge of the GIP system, carriers of dysfunctional GIP variants would be expected to be at risk of reduced pancreatic endocrine and bone tissue functions as well as affected lipid metabolism. Missense variants leading to pathological states can now be identified in large population studies that include exome and genome data (38, 39). The completion of the human genome project followed by large-scale sequencing projects, such as the 1000 Genomes project (40) and Genome Aggregation Database (gnomAD) (21) has enabled studies of genetic variants and associated phenotypical traits (41). While focus has been placed on prominent drug targets among GPCRs (42), little attention has been given to the genetic variability of peptide hormones in humans, which include more than 340 secreted forms, most of which target GPCRs (43, 44).

With 14.9 million unique exome variants identified in 125,748 human exomes (gnomAD) and even more to be discovered, this large number of genetic variants exceeds what experimentally can be investigated using *in vitro* and *in vivo* based techniques (21). Given the number of variants and the technical constraints, computational analysis and predictions have emerged as important tools to evaluate the putative impact of genetic alterations. Indeed, prediction models have shown promising concordance with experimental data, especially deep-mutational scanning data (MAVE) (45–47). Hence, computational models can provide valuable information allowing selection of specific variants for more detailed and comprehensive *in silico* and/or experimental investigations.

Diabetes is one of the leading causes of premature mortality with an estimated prevalence of 570 million individuals by 2025 worldwide. Thus, the burden of diabetes calls for development of new and efficacious therapies (48). Several drugs indicated for the treatment of T2D, and obesity mimic the action of GLP-1 (49), the incretin sister hormone of GIP. Hence, GIP holds a promising therapeutic potential, as supported by the results of clinical trials with dual GLP-1R/GIPR agonists such as tirzepatide (50). However, the apparent lack of GIP efficacy in patient with T2D coupled with the obesity protective phenotype of GIPR knockout mice, initially discouraged the development of GIPR agonists for diabetes therapy and even suggested that development of GIPR antagonists might be expedient. This agonism *vs*. antagonism confusion has been rekindled with recent data showing that GIPR antagonizing antibodies as well as long-acting GIP agonists provide notable reduction in body weight and improvements of glucose control when combined with GLP-1R agonists in preclinical models (51, 52). Interpretation of naturally occurring variants in the GIP peptide may provide valuable inputs to this debate by expanding our understanding of structural and functional features of the GIP system. Thus, the elucidation of GIP peptide variants can provide a causal link between genotypes and phenotypes, ultimately contributing to translational interpretation of data and assessment of potential treatment modalities. Here, we investigate the spectrum of missense variants in the GIP gene based on exome and genome data from three independent cohorts and discuss the findings in conjunction with known experimental data with a view to further elucidate the structure-function relationships and their physiological consequences.

## Results

### Spectrum and Prevalence of Missense Variants in the GIP Precursor

To elucidate the spectrum of mutations in the GIP gene, we aggregated human exome and genome sequence data from three diverse and independent cohorts: UK Biobank (20), (gnomAD) (21), and Trans-Omics for Precision Medicine (TOPMed) (22) collecting exome and genome sequences from 721,991 individuals. We identified 168 unique amino acid-changing missense variants in the GIP gene at 105 distinct amino acid positions covering 69% of the entire sequence stretch (**Figure 1A**). The distinct pre-pro-GIP regions had different variant densities (number of positions containing a variant divided by the peptide length) ranging from 57% in the GIP peptide: GIP(1-42)) to 79% (in the N-terminal propeptide).

Among the 168 missense variants, we identified 97 in 454,787 exomes from the UK Biobank (20), 81 missense variants in the gnomAD (125,748 exomes and 15,708 genomes) (21), and 84 missense variants in the TOPmed database (132,345 genomes) (22) (**Figure 1B**). The mean minor allele frequency (MAF) of variants was generally higher when the variant was found in more than one cohort, with mean MAF of 0.019, 1.89·10^-5^, and 4.76·10^-6^ when detected in all three, two or single cohorts, respectively. The most common variant S103G (see **SI Table 1** for transcript positions and nucleotide changes), located in the C-terminal propeptide, displayed an AF of 0.68 in the UK Biobank cohort. In contrast, singletons, i.e., variants identified in one individual only, were scattered across the entire pre-pro-GIP peptide.

Next, we assessed the GIP(1-42) sequence and found 41 missense variants affecting 24 amino acid positions (57%) (**Figure 1C**). The most frequent variant, L27V, in GIP(1-42), exhibited an MAF of 1.55·10^-4^, whereas the mean MAF of variants in GIP(1-42) was 1.75·10^-5^.

### Missense Variants in the Biologically Active Peptide GIP(1-42) Are Predicted to be More Deleterious

**Figure 2:**
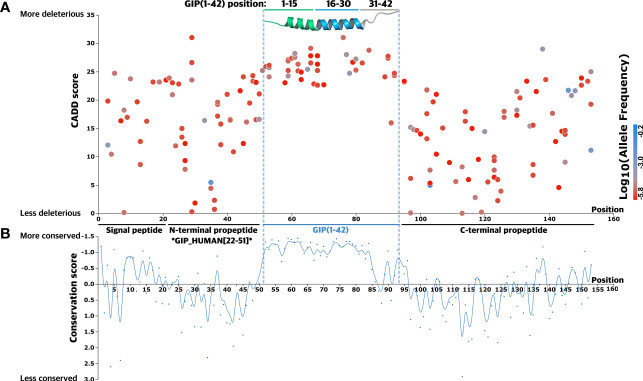
Mutational spectrum of GIP gene missense variants. **(A)** A total of 168 glucose-dependent insulinotropic polypeptide (GIP) gene missense variants were mapped according to their amino acid position. A Combined Annotation-Dependent Depletion (CADD) score, predicting variant deleteriousness, was employed to establish a link between mutations and regions of particular importance in the precursor hormone. The GIP peptide is highlighted in blue and visualized by an experimental 3D structure (PDBid: 2OBU) (53), N-terminal segment (green), core segment (blue), C-terminal segment (grey). The corresponding variant allele frequency is colored by a gradient scale with more frequent variants (blue) and less frequent variants (red). **(B)** The bottom graph displays the conservation score at each alignment positions as obtained from ConSurf (54) to determine functionally important segments of the precursor hormone. A lower conservation score displays more conserved amino acid sites, indicating the GIP peptide sequence is more conserved across species (all metazoan, including invertebrates).

We mapped all 168 missense variants along with their amino acid position in the pre-pro-GIP sequence. Next, we employed a Combined Annotation-Dependent Depletion (CADD) score, which predicts the deleteriousness, to delineate functional consequences of individual variants (**Figure 2A**). The CADD model is based on a machine learning model that integrates information into a single score based on more than 60 different annotations such as conservation-based and functional metrics. Moreover, the CADD model is normalized and has been applied to all potential 8.6 billion single-nucleotide variants in the human genome. The final score is scaled where 0-10 is given to the 90% least deleterious variants, 10-20 applies to the top 10% most deleterious variants, and a score of 20-30 reflects the top 1% most deleterious, etc. (45, 46).

The mean CADD score for variants in the pre-pro-GIP was 17.7, with scores spanning 0.035 to 31. The variant W76G, at position 25 in the GIP(1-42) peptide, exhibited the highest CADD score of 31. In contrast, V113A, located in the C-terminal propeptide, demonstrated the least pronounced CADD score of 0.035. The highly deleterious variant W76G was only found in one data set (TOPMed), whereas the benign variant, V113A, was present in two data sets (TOPMed and gnomAD). This observation is in accordance with the GIP(1-42) peptide, which showed the lowest mean MAF of 1.7·10^-5^ compared to the C-terminal propeptide (mean MAF of 9.8·10^-3^), suggesting there is stronger evolutionary pressure for variants in the GIP(1-42) peptide (**Figure 2A**).

Next, we introduced a conservation score (CS) to substantiate the importance of the different segments and amino acid sites within the pre-pro-GIP sequence (**Figure 2B**). The degree of conservation is determined by the evolutionary rate at each alignment position, varying from more conserved sites, evolving at a slower rate, and vice versa (55, 56). Here, the individual pre-pro-GIP regions showed considerable variation in conservation, with a remarkable conservation of the GIP(1-42) sequence.

### The GIP(1-42) Peptide Displays a Significantly Higher Evolutionary Conservation

**Figure 3:**
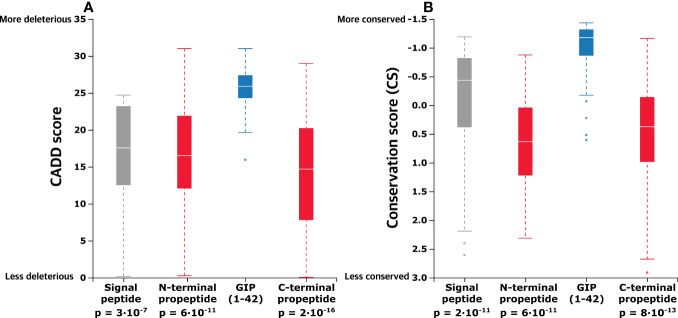
In the proGIP gene, the coding region of the biologically active peptide is more conserved, and missense variants here are more deleterious. **(A)** Aggregated mean CADD scores (abbreviation of Combined Annotation-Dependent Depletion, predicting variant deleteriousness) for individual pre-pro-GIP regions demonstrate that variants in the GIP sequence exhibit a significantly higher mean CADD score than those of the other peptide regions. **(B)** Comparison of aggregated mean conservation scores (CS) indicates stronger evolutionary conservation relative to the other regions of the GIP gene central lines indicates median. Statistical significance between samples was assessed using the Mann-Whitney U test.

We aggregated the obtained CADD scores across the entire pre-pro-GIP sequence to elaborate on the functional analysis and capture potential differences. Thus, we compared the neighboring pre-pro-GIP segments with the GIP(1-42) peptide as this region demonstrated the highest mean CADD score. This revealed that the mean CADD score of all variants in the GIP(1-42) peptide was significantly higher than those of the signal peptide (Mann-Whitney U test: p-value = 2.98·10^-7^), the N-terminal propeptide (p-value = 6.25·10^-11^), and the C-terminal propeptide (p-value = 2.22·10^-16^) (**Figure 3A**).

Furthermore, we aggregated the CS to compare mean differences between the distinct pre-pro-GIP segments. Again, the GIP(1-42) peptide was used as a reference due to the highest degree of conservation, reflected by the lowest CS. This showed that the GIP(1-42) (mean CS –0.946) is significantly more conserved than the rest of the pre-pro-GIP segments; signal peptide (mean CS -0.039; p-value: 2·10^-5^), N-terminal propeptide (mean CS 0.636, p-value: 6·10^-11^), C-terminal propeptide (mean CS 0.431; p-value: 8·10^-13^) (**Figure 3B**). This analysis indicates that GIP(1-42) is more conserved, and variants in this region are potentially more pathogenic than the rest of the pre-pro-GIP regions.

### Mapping Conserved Positions and Detrimental Variants Within the GIP(1-42) Peptide

We focused on the 41 genetic variants found in GIP(1-42) to dissect different peptide regions as they exhibit differential features with respect to receptor interaction. Taking the two-domain binding mechanism into account (35), GIP(1-42) was divided into three segments: a N-terminal segment (residue 1-15), a core segment (residue 16-30), and a C-terminal segment (residue 31-42).

**Figure 4:**
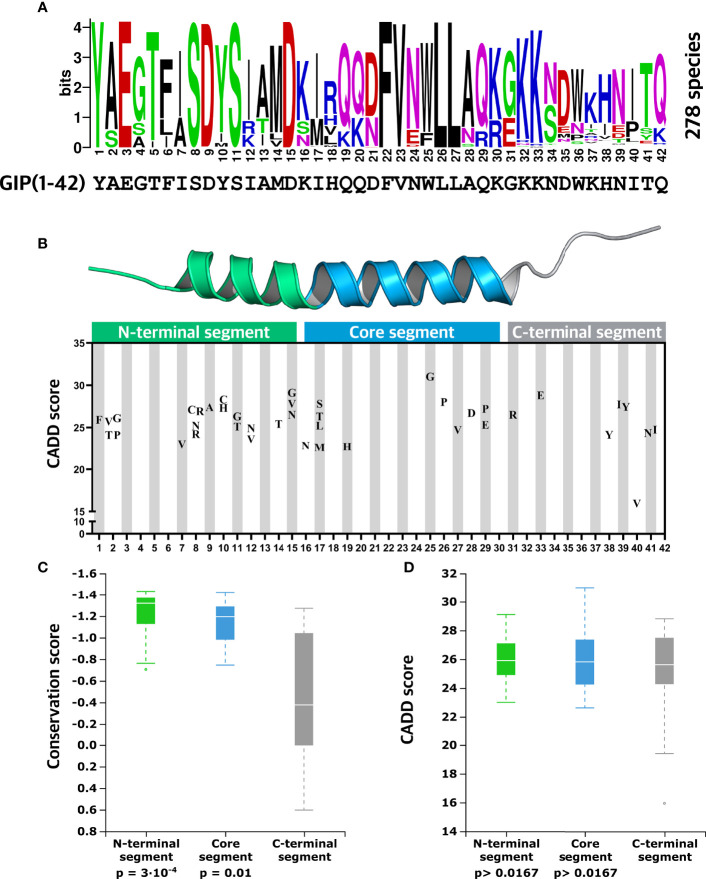
Evolutionary insights into essential GIP peptide positions. **(A)** Amino acid conservation logo plot determined by multiple sequence alignment using GIP sequences from 278 different species. The overall height over the letter stack indicates sequence conservation in the specific position, whereas the height of each letter indicates the relative frequency of each amino acid at that specific position. **(B)** Distribution of missense variants in the GIP peptide is displayed along with predicted deleteriousness using the CADD (Combined Annotation-Dependent Depletion) score. **(C)** Comparisons of mean conservation score between the GIP peptide segments show that the N-terminal segment (positions 1-15) and core segment (positions 16-30) display a significantly higher degree of conservation compared to the C-terminal segment. **(D)** Aggregated mean CADD scores (abbreviation of Combined Annotation-Dependent Depletion, predicting variant deleteriousness) for individual GIP peptide segments show that variants in the GIP sequence exhibit similar mean CADD score independent of the segment they are located in. Statistical significance between sample means was assessed using the Mann-Whitney U test (p-value threshold 0.0167).

First, to elucidate the conservation of specific positions and segments in GIP(1-42), we employed a multiple sequence alignment including 278 species. In the N-terminal segment (1-15), positions (1,3,5,8-11, and 15) displayed a high degree of conservation across species (**Figure 4A**). In the core segment (16-30), the hydrophobic positions 22,23,26, and 27, complementing a binding groove in the GIPR (57), showed the highest degree of conservation (CS < -1.328). The C-terminal segment (31-42) represents the C-terminal tail, which is unique for GIP(1-42) and structurally less ordered than the closely related class B1 peptides (58). Positions in this segment showed the lowest degree of conservation, except positions 32 and 33, located in the PC2 cleavage motif (G31; K32; K33) (59) (**Figure 4A**). Throughout evolution, some species lack part of or the entire C-terminal segment of the GIP(1-42) peptide, with lengths of GIP varying from 29 amino acids in fish to 42 amino acids in humans (60). This is interesting in view of the fact that the GIP(1-42) and GIP(1-30) peptides have identical biological activity towards the human GIPR.

Since GIP(1-42) exhibited significantly higher CADD scores than the neighboring pre-pro-GIP regions, we mapped GIP(1-42) missense variants along with their CADD scores (**Figure 4B**). In the N-terminal segment (1-15), we observed 21 missense variants with a mean CADD score of 25.9, meaning that alterations in this segment are likely to have a damaging effect, in keeping with the findings that this region of the peptide has essential interactions with the receptor binding pocket. Among these, we discovered Y1F in the highly conserved position 1 with a CADD score of 25.9. In position 2, usually containing an alanine recognized by the proteolytic enzyme DPP-4 (24), we identified four variants with CADD scores of 24.1-26.1. Interestingly, no variants appeared in positions 3-6. The three positions with the highest degree of conservation in the N-terminal segment (1-15), positions 8, 9, and 11, contained four, one, and two variants, respectively, with CADD scores ranging from 24.2 to 28.2. Among the variants in the N-terminal segment, D15G showed the highest CADD score of 29.1.

In the core segment (**Figure 4B**), we observed five variants in positions 16 (one variant) and 17 (four variants) with high CADD scores of 22.8-27.8, despite less evolutionary conservation at these positions. In contrast, no variants were present in positions 18, 20-24, and only one was found in position 19. Six variants were identified between positions 25-29 with relatively high CADD scores of 24.7-31, including variants in the hydrophobic positions 26 and 27, known to interact with resides in the extracellular domain (ECD) of the GIPR (57). Moreover, the variant W25G showed the most prominent CADD score of 31 among all GIP variants. In the C-terminal segment, two detrimental variants, G31R and K33E, were found in the PC2 motif with CADD scores of 26.5-28.8 (59) (**Figure 4B**). Six variants were observed between residue 38 to 41, including I40V predicted to be the least harmful GIP(1-42) variant with a CADD score of 15.9 (mean CADD score of all pre-pro-GIP variants; 17.7).

Last, to explore differences in conservation between the three distinct GIP(1-42) segments, we compared the mean CS for the N-terminal segment (mean CS; -1.23), core-segment (mean CS -1.07), and C-terminal segment (mean CS -0.43), using a Mann-Whitney U test (**Figure 4C**). We corrected our significance threshold to 0.0167 to account for the number of comparisons. This revealed that the N-terminal segment (p-value = 3·10^-4^) and core segment (p-value = 0.011) are significantly more conserved than the C-terminal segment. To substantiate the observations regarding segment conservation, we assessed the mean CADD scores for the N-terminal segment (mean CADD 25.9), core segment (mean CADD 25.9), C-terminal segment (mean CADD 24.9) using the same statistical approach (**Figure 4D**). Surprisingly, there were no significant differences between the mean CADD scores for the three GIP(1-42) segments (p-value > 0.0167).

To substantiate the predicted impact, we employed an additional prediction model, the evolutionary model of variants effect (EVE). We then compared CADD scores with the obtained EVE scores for GIP(1-42) variant, revealing that the two variant effect scores correlated strongly (Pearson’s correlation; r = 0.645 and P = 5.31·10^-6^). Moreover, for both EVE and CADD, the deleteriousness of variants between the three GIP(1-42) segments were similar. Collectively, this suggests that variants in all the different GIP(1-42) segments, in general, are highly detrimental, despite diverse segment conservation and distinct functional roles.

## Discussion

Genome and exome sequencing of large cross-population cohorts have enabled studies of rare genetic variants at epidemiological scale (61), not possible with chip-based genotyping methods which are more suitable for common variants (39, 62). Here, by combining data from three independent cohorts comprising 721,991 exomes and genomes, we identified 168 missense variants (167 with MAF <0.5%) scattered across the human GIP gene. Analyzing variant distribution across the GIP transcript sequence, we observed higher frequency of variants located outside of the mature GIP peptide sequences, illustrating an evolutionary conservation of this peptide hormone, and revealing sequence motifs important for its structure and function. To elucidate potential deleteriousness of missense variants, we selected both the CADD and EVE methods as representative variant effect predictors among a long array of published prediction models (63). This analysis revealed that variants in the mature, circulating GIP(1-42) are significantly more deleterious than those in the surrounding segments of the pre-pro-peptide.

### Variants in the N-Terminal Segment of Importance for Receptor Activation

**Figure 5:**
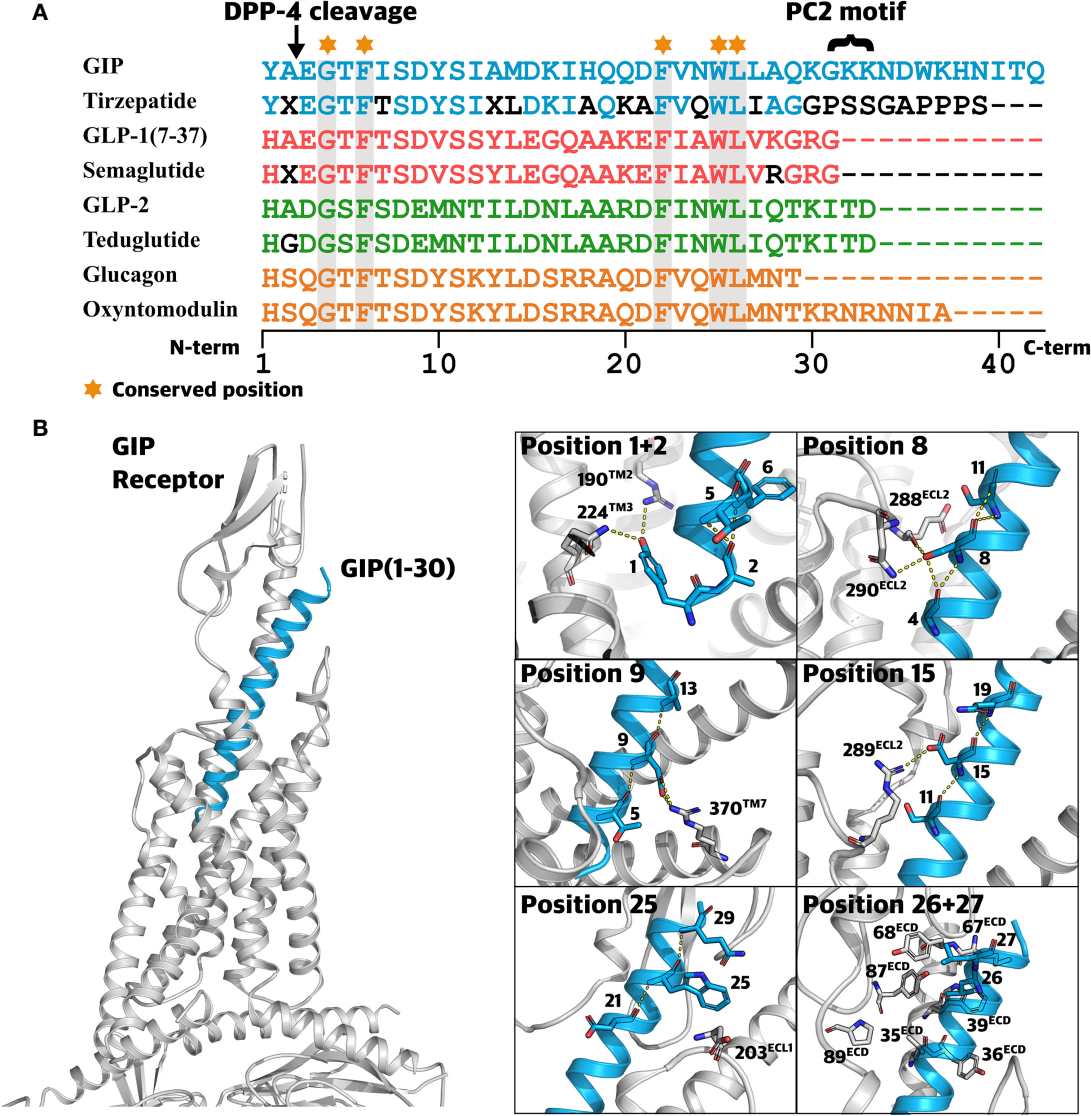
Class B1 ligands and the structural perspective. **(A)** Sequence alignment of related class B1 ligands and peptide analogs. Sequence alignment of glucose-dependent insulinotropic polypeptide (GIP), tirzepatide (dual GIPR/GLP-1R agonist), glucagon-like peptide-1 (GLP-1), semaglutide (GLP-1 analog), GLP-2, teduglutide (GLP-2 analog), glucagon, and oxyntomodulin. A ‘X’ denotes α-aminoisobutyric acid (Aib). Amino acids displayed by black letters represent sites deviating from those of the endogenous peptide. Conserved positions across all peptides are highlighted in grey. **(B)** Highlighted ligand-receptor interactions between GIP and the GIPR. 3D visualization of GIP(1-30) (blue) in complex with the GIPR (grey) and the Gα_s_ protein (grey) (Protein Data Bank: 7DTY) (57). Frames to the right display important hydrophobic interactions and polar interactions (yellow dotted lines) between GIPR (grey) and GIP (blue).

Since docking of GIP N-terminus into the receptor’s transmembrane domain initiates conformation rearrangements necessary for GIPR activation (35) and N-terminal truncations of the GIP peptide cause loss of its agonistic properties, alterations in this segment are likely to disrupt the ligand-mediated receptor activation (6). Although speculative, it may even be possible that N-terminal mutations result in partial agonists or even antagonists. Among all 15 residue positions in the N-terminal GIP segment, ten were found to be altered by rare genetic variations. Position 1 is involved in several interactions with GIPR residues (e.g., R190^2.67^ and Q224^3.37^, Wootten numbering (64)) promoting GIPR activation (57, 65) (**Figure 5**), and consistent with this, removal of the first amino acid of the full agonist GIP(1-30)NH_2_ causes a remarkable decrease in potency (6). Moreover, alanine substitution of position 1 also severely decreases potency and diminishes insulinotropic activity (66, 67). Similarly, alanine substitutions at this position in glucagon and GLP-1 also severely impact functionality, emphasizing the importance of position 1 for receptor activation in this peptide hormone family (68, 69). Hence, receptor activation is most likely hampered by mutations in position 1, taking the essential role of this position and previous investigations into consideration.

Interestingly, even among the ~720,000 individuals, no variants were present between positions 3-6 suggesting that such variants are highly unfavorable. Supporting this, engineered alanine substitutions of residue 3 and 5 lead to >10-fold reduction of potency (67) and the truncated GIP(3-30)NH_2_-5-30)NH_2_ loses the ability to activate the GIPR. Among all N-terminal truncations, from GIP(1-30) to GIP(9-30), removal of the first five (in GIP(6-30)NH_2_) results in the most drastic decrease in potency and affinity, suggesting that this truncation impairs the ligand stability, potentially bringing residue 6 into an energetically unfavorable conformation for binding (6), i.e., disrupting ligand-receptor complex formation. In line with this, alanine substitution of residue 6 led to reduced stimulation of insulin secretion (66).

Positions 7-15 are essential for receptor activation, as previously described in structure-activity investigations involving truncations of GIP from GIP(7-30)NH_2_ to GIP(15-30)NH_2_. Here, removal of the first 7-15 amino acid of GIP(1-30)NH_2_ promoted antagonistic properties of the truncated peptide (6, 70, 71). For GIP(1-42), the substitution with alanine in positions 7, 8 or 15 created partial agonists, supporting these positions as important for receptor activation (6, 71, 72).

The serine in position 8 is the most conserved across species (CS of -1.433) and has been shown to contribute to several interactions with GIPR residues including R289^ECL2^ and N290^ECL2^ (**Figure 5**) (57, 67). Peptide truncations and alanine substitutions have unraveled the importance of position 8 reflected by decrease in potency for cAMP formation and insulinotropic activity (6, 67, 73). In this study, S8C, S8N, S8R(A/T), and S8R(T/G), displayed CADD scores ranging from 24.2 to 27.1, indicating this position as vulnerable to a variety of amino acid alterations.

The highly conserved residue D9 (CS -1,40) is involved in several interactions with the GIPR, including hydrogen bond formation with residue R370^7.35^ (57) (**Figure 5**). Disruption of this interaction appears to affect receptor activation, in line with a 4- and 35-fold decrease in potency for D9A, a mutation naturally occurring in the human population (57, 67). Likewise, the substitution of conserved tyrosine to an alanine in position 10 caused a 107-fold reduction in potency (67), consistent with the high predicted deleteriousness for both Y10H and Y10C. These results support the pivotal role of the N-terminus for receptor activation. Hence, we expect genetic variants occurring in the N-terminal segment (positions 1-15) to affect physiological functions resulting from diminished signaling properties of GIP(1-42).

### Variants in the Core Segment Central for Initial Receptor Binding

The ECD of the GIPR recognizes and interacts with the core segment of GIP, which adopts an alpha-helical conformation similar to other class B1 receptor ligands (58, 66). We identified 12 missense variants in this segment in eight different positions. Interestingly, no variants were observed in positions 20-24, including position 22 which is highly conserved among all related ligands, indicating that this motif is important for the interaction with GIPR (57) (**Figure 5**).

Consistent with the lowest conservation score for residue I17, four genetic variants were identified in this position (I17L, I17M, I17S, and I17T). Nonetheless, variants affecting this position still displayed relatively high CADD, an observation supporting the proposed interaction between I17 and the GIPR residue L35^ECD^, contributing to the stabilization of the GIP/GIPR complex (74).

Consistent with a 100% conservation of position 25 across all related class B1 receptor ligands, W25G had the highest predicted deleteriousness among all GIP peptide variants (CADD score: 31). This is consistent with this position being conserved across all related class B1 receptor ligands (**Figure 5**). Structural data from molecular dynamic simulations support its importance and indicate the formation of a weak hydrogen bond between W25 and the GIPR residue D203^ECL1^ (67) (**Figure 5**). Thus, mutating this residue can disrupt the initial interaction between the GIP(1-42) and the receptor ECD, primarily attributed to hydrophobic interactions in the defined core segment in the middle (73).

Complementing a series of hydrophobic residues in the ECD of the GIPR, the binding of GIP(1-42) is accompanied by hydrophobic interactions with the highly conserved GIP(1-42) residues 22, 23, 26, and 27 (58) (**Figure 5**). Thus, the genetic variants L26P and L27V might cause destabilization of this interaction and alter the binding profile.

### Variants in the C-Terminal Unstructured Segment

Until the discovery of GIP_HUMAN(22-51) (29), GIP(1-42) had long been established as the only hormone to be encoded by the GIP gene (**Figure 2**), contrasting to the many structurally related proglucagon-derived peptides arising from the GCG gene (75). Moreover, the GIP peptide distinguishes itself from the related class B1 receptor ligands by having an extended C-terminal segment (**Figure 5**). This segment is proposed to have a disordered secondary structure, neither involved in receptor binding nor activation (58). However, the segment is postulated to provide structural stability in aqueous solvent (66). Interestingly, in some species, the C-terminal segment of GIP(1-42) is truncated or completely absent (60). Despite this segment being less evolutionary conserved, variants in that part of GIP appear detrimental with a mean CADD score of 24.9, not remarkably different from the two other segments (mean CADD; N-terminal segment 25.9 and core segment 25.9).

However, previous *in vivo* studies have demonstrated a full agonist activity regarding insulin secretion with the truncated GIP(1-38) and GIP(1-39) (76, 77), questioning the importance of residue 39 and the predicted deleteriousness.

Emphasizing the absence of the C-terminal tail to enhance antagonistic properties of N-terminally truncated GIP peptides, *in vitro* characterization showed that GIP(3-30)NH_2_ has a 26-fold higher inhibitory potency than GIP(3-42) (6). However, in another study, the presence of the C-terminal tail resulted in partial agonism of N-terminally truncated peptides: GIP(3- to 8-42) (72). Previously, the C-terminal tail has been suggested to play a role in the structural stability of GIP (66), but this role is far from being understood. Thus, it is challenging to interpret the effect of mutations in this segment.

### Impact of GIP(1-42) Variants on Pharmacokinetics

The amino acid in position 2 plays a crucial role in the short half-life of GIP (T_1/2_: 7 minutes) (19) as this is part of the recognition site for DPP-4 where an alanine or proline in position 2 results in cleavage between positions 2 and 3 into the inactive metabolite GIP(3-42) (24). We identified four variants in position 2: A2V, A2T, A2P, and A2G. Of these, individuals carrying A2V, A2T, and A2G could have a prolonged half-life of GIP due to reduced DPP-4 degradation. Several therapeutics from this family of peptides have amino acid substitutions in position 2 to protect from DPP-4 degradation and thereby increase their half-life. This is for instance the case for the GLP-2 analog teduglutide (**Figure 5**), with a glycine in position 2, which has a half-life of 2 hours compared to 7 minutes for endogenous GLP-2 (alanine in position 2) (78). Although these two half-life determinations were done subcutaneously and intravenously, the difference points towards increased half-life for Teduglutide. Of the identified residues in position 2, individuals carrying the variants A2V, A2T and A2G could therefore produce a GIP molecule with prolonged half-life due to reduced DPP-4 degradation.

Another two variants, G31R and K33E, were found in an area linked to pharmacokinetic properties, the PC2 cleavage motif (G31;K32;K33) (59). Thus, mutations in this motif could disrupt the PC2 cleavage site, leading to reduced GIP(1-30)NH_2_ levels and higher circulating levels of GIP(1-42) in individuals carrying these variants. Taken together, the interpretation of genetic variants should not exclusively rely on altered receptor-ligand interactions but also consider variants in key positions for the hormone’s metabolism and clearance.

### Link to Phenotypic Traits By Alterations in the GIP/GIPR System

Given the individual exome data for the 450k exome-sequenced UK Biobank participants, we identified 185 individuals with heterozygous missense mutations in their GIP peptide sequence. This is remarkably few, considering the 24 distinctly mutated positions in the 42 amino acid-long sequence. This low GIP peptide diversity may indicate high evolutionary constraints on phenotypic consequences on random mutations within the GIP peptide. Interestingly, the most frequent GIP missense variants reside at position 2. Heterozygous carriers of either the Gly or Val variant display a slightly lower mean BMI (n=76; 27.12 kg/m^2^) than the mean BMI across all other GIP missense variant carriers (n=91; 27.74 kg/m^2^; no correction or statistical test performed). This could indicate that a more prolonged GIP action, given the likely reduced DPP-4 degradation, has long-term consequences on weight. Further studies need to be conducted to elucidate the specific effects on metabolic outcomes including disease risks for the range of GIP missense mutations. This undoubtedly requires much larger cohort sample sizes given the low number of GIP variant carriers.

While the current study delineates the potential impact of genetic variants in the GIP gene and resulting amino acid sequence, it also has several limitations. The variant prediction models employed in this study are primarily based on sequence conservation across a limited set of species. Structural aspects, such as peptide-protein interactions need to be considered to gain insights into elements involved in crucial interaction with receptor residues. Free binding energy calculations could substantiate the results of computational models trained on evolutionary data utilized in this study (79). However, the full-length structure of GIP has not been resolved due to the disordered structure of the C-terminal tail (position 31-42) (57). Missense variants found in the GIP peptide might also affect the cross-talk between receptors and may even bias/shift the activation toward a different signaling pathway. Thus, the impact of missense variants needs to be further examined in specific and sensitive *in vitro* experiments as well as translational studies. Therefore, the framework described herein should be treated as hypothesis generating. Although this study focused on amino acid-changing missense variants, receptors and peptide-ligands can also harbor other types of variants, for instance, intron and synonymous variants, which can influence transcription efficacy and trafficking and ultimately alter circulating hormone levels (80). Moreover, variants in the coding region are the primary focus of the study. However, 96.4% of all variants reside in non-coding regions (81). Other factors to consider when interpreting the significance of genetic data are buffering mechanisms such as epistatic interaction, allele-specific interaction, and heterozygous variants, which can alleviate the direct effect of a given variant (82, 83). Although efforts are made to generate large-scale information on genetic variation, the relatively small cohort size and population diversity utilized in this study impact which rare variants we identify. In the future, we hope to incorporate more comprehensive and diverse population data from sequencing efforts, such as the +1 Million Genomes initiative, FinnGen, and The Estonian Biobank (84–86).

The unique methodological framework presented in this study is applicable to other hormone precursor genes and their cognate receptors to aid the understanding of essential structural elements and peptide-receptor structure-function relationships. Grouping genetic variants with similar functional characteristics can be employed in personalized drug regimens, e.g., utilizing variants with a prolonged/decreased half-life in the treatment of phenotypes with altered peptide metabolism. The better linkage between genotypes and phenotypes could ultimately aid the discovery of new drug targets such as the gain-of-function variants for MCR4 described by Lotta et al. which are associated with a decreased risk of obesity (87).

Elucidating the impact of genetic variations on the GIP endogenous system (88), accompanied by structural insights into the peptide bound GIPR (57, 89), can provide valuable information for future drug discovery efforts. This is highlighted by the fact that both agonists and antagonists at the GIPR could provide valuable modes of action in the treatment of T2D and obesity (90). Recently, two GIPR mutations have been shown to link to lower BMI in carriers (37). Following a deep molecular characterization both mutations displayed reduced Gα_s_ protein coupling as well as impaired β-arrestin 2 recruitment and internalization. Similarly, persons with rare GIP mutations might have improved glucose metabolism and fat deposition properties that could inspire further analogue modifications. In conclusion, the 168 missense variants identified in this work may facilitate the *in vitro* characterizations of GIP variants which can be helpful in the stratification and selection towards effective clinical translation.

## Methods

### Compiling of the Genetic Dataset

We aggregated human exome and genome sequence data from three independent and diverse cohorts focusing on missense variants but disregarding other mutations occurring in the GIP gene upon nucleotide changes such as nonsense mutations, splicing mutations etc. (91). First, we took advantage of all individual level exome information from 454,787 individuals in the UK Biobank, from which we identified 97 missense variants in the GIP gene. The UK Biobank is a large-scale biomedical database and resource providing in-depth health information about approximately 500,000 participants with exome sequence information for 454,787 individuals; importantly, all participants have given general consent for health-related research (20). We identified the GIP gene in region 17:47035916-47045958 using genome build GRCh38. We used ENST00000357424.2 as canonical transcript and P09681 (GIP_HUMAN) as UniProt identifier.

Secondly, from gnomAD v.2.1.1 (https://gnomad.broadinstitute.org/), we identified 81 missense variants in the GIP gene. The gnomAD contains whole-exome and whole-genome sequence data aggregated from human sequencing projects spanning six global and eight sub-continental ancestries and includes 125,748 whole-exomes and 15,708 whole-genome sequences from a total of 141,456 unrelated individuals (21).

Lastly, we integrated data from TOPMed Freeze 8 on GRCh38 (https://topmed.nhlbi.nih.gov/), from which we identified 84 missense variants in the GIP gene. This database comprises >80 studies containing 132,345 whole-genome sequences from approximately 180,000 participants with ancestral and ethnic diversity (22).

### Conservations Scoring and Calculations of Predicted Deleteriousness

We employed the Combined Annotation-Dependent Depletion (CADD) method to score and assess variants in the GIP gene based on their potential to be pathogenic. The CADD score is built from more than 60 genomic features and normalized to approximately all 8.6 billion variants across the genome. The CADD score is a scaled score where a score of 0-10 reflects the 90% least deleterious variants, a score of 10-20 reflects the top 10% most deleterious variants, and a score of 20-30 reflect the top 1% most deleterious variants among all 8.6 billion potential genetic variants.

CADD scores have been retrieved for all missense variants from the CADD Web API (https://cadd.gs.washington.edu/api) (92) after lifting all variant positions from GRCh37 to the GRCh38 reference assembly using Ensemble’s assembly map (https://rest.ensembl.org/documentation/info/assembly_map).

To substantiate the interpretation of variant deleteriousness, we employed a state-of-the variant effect predictor, Evolutionary model of variants effect (EVE). The model is trained on the tendency of variants to be pathogenic based on the distribution of sequence variation across species. The EVE model yields an EVE score ranging from 0 to 1. A score of zero reflects the most benign variant and one reflects the most pathogenic variant. Evolutionary model of variant effect (EVE) scores have been retrieved from (https://evemodel.org/) (47).

We furthermore employed a conservation score with the rationale that sequence conservation across species can yield insight into the consequences of sequence diversity within species. Conservation scores have been extracted from (https://consurf.tau.ac.il/) and calculated using the Bayesian method with standard parameters (54). The GIP protein sequence in FASTA format was obtained from (https://www.uniprot.org/uniprot/P09681). The most conserved amino acid positions are reflected by the lowest conservation score.

The Logo plot was generated by (https://weblogo.berkeley.edu/) (93), using GIP orthologue alignments of 278 species sourced from Ensembl release 105 (94). Statistical significance was addressed by a Mann-Whitney U test evaluating the range of CADD scores and conservation scores between the various GIP segments.

### 3D Visualization and Peptide Alignments

3D representation of the GIP(1-30) in complex with the GIPR (Protein Data Bank: 7DTY) (57) was visualized by PyMOL (http://www.pymol.org/pymol). The cryo-EM structure 7DTY is an active-state structure of the GIP receptor in complex with GIP and a G_s_ heterotrimer at a global resolution of 2.9 Å. One letter amino acid alignments of class B1 peptides and peptide analogs were generated using the Interactive Tree of Life (iTOL) (https://itol.embl.de/) (95). Bubble chart, line chart, and box plots were visualized using RAWGraphs 2.0 (https://rawgraphs.io/) (96).

## Data Availability Statement

The datasets presented in this study can be found in online repositories. The names of the repository/repositories and accession number(s) can be found in the article/**Supplementary Material**.

## Author Contributions 

Conceptualization: PL, AH, and MR. Methodology: PL and AH. Validation: PL, AH, and MR. Formel analysis: PL and AH. Investigations: PL, LG, and AH. Resources: PL, LG, AH, and MR. Data curation: PL and AH. Writing – original draft: PL. Writing – review and editing: PL, LG, JM, JH, AH, and MR. Visualization: PL. Supervision: LG, AH, and MR. Project administration: PL, AH, and MR. Funding acquisition: LG, AH, and MR. All authors contributed to the article and approved the submitted version.

## Funding

LG is supported by the BRIDGE – Translational Excellence Programme (bridge.ku.dk) at the Faculty of Health and Medical Sciences, University of Copenhagen, funded by the Novo Nordisk Foundation grant agreement no. NNF18SA0034956. AH would like to gratefully acknowledge funding from the Lundbeck Foundation (R278-2018-180). MR received funding from the Novo Nordisk Foundation (NNF21OC00671) and (NNF21OC0070347), from a donation from deceased Valter Alex Torbjørn Eichmuller (VAT Eichmuller)-2020-117043 and from Kirsten and Freddy Johansens Foundation (KFJ)-2017-112697.

## Conflict of Interest

Author JM was employed by the company Novo Nordisk Research Center Indianapolis. Author JH was a member of the advisory boards at NovoNordisk; co-founder and member of Board of Antag Therapeutics and Bainan Biotech. Author MMR was Co-founder of Antag Therapeutics and Bainan Biotech. Chairman of Board of Bainan Biotech.

The authors declare that the research was conducted in the absence of any commercial or financial relationships that could be construed as a potential conflict of interest.

## Publisher’s Note

All claims expressed in this article are solely those of the authors and do not necessarily represent those of their affiliated organizations, or those of the publisher, the editors and the reviewers. Any product that may be evaluated in this article, or claim that may be made by its manufacturer, is not guaranteed or endorsed by the publisher.
